# Long term follow up after surgery in congenitally corrected transposition of the great arteries with a right ventricle in the systemic circulation

**DOI:** 10.1186/1749-8090-5-74

**Published:** 2010-09-28

**Authors:** Ad JJC Bogers, Stuart J Head, Peter L de Jong, Maarten Witsenburg, Arie Pieter Kappetein

**Affiliations:** 1 Department of Cardiothoracic Surgery, Erasmus University Medical Center, PO Box 2040, 3000 CA, Rotterdam, The Netherlands; 2 Department of Paediatric Cardiology, Erasmus University Medical Center, PO Box 2040, 3000 CA, Rotterdam, The Netherlands; 3 Department of Cardiology, Erasmus University Medical Center, PO Box 2040, 3000 CA, Rotterdam, The Netherlands

## Abstract

**Aim of the study:**

To investigate the long-term outcome of surgical treatment for congenitally corrected transposition of the great arteries (CCTGA), in patients with biventricular repair with the right ventricle as systemic ventricle.

**Methods:**

A total of 32 patients with CCTGA were operated between January 1972 and October 2008. These operations comprised 18 patients with a repair with a normal left ventricular outflow tract, 11 patients with a Rastelli repair of the left ventricle to the pulmonary artery and 3 patients with a cardiac transplantation.

**Results:**

Excluding the cardiac transplantation patients, mean age at operation was 16 years (sd 15 years, range 1 week - 49 years). Median follow-up was 12 years (sd 10 years, range 7 days - 32 years). Survival obtained from Kaplan-Meier analysis at 20 years after surgery was 63% (CI 53-73%). For the non-Rastelli group these data at 20 years were 62% (CI 48-76%) and for the Rastelli group 67% (CI 51-83%). Freedom of reoperation at 20 years was 32% (CI 19-45%) in the overall group. In the non-Rastelli group the data at 20 years were 47% (CI 11-83%) and for the Rastelli group 21% (CI 0-54%) after almost 19 years.

**Conclusions:**

Long term follow up confirms that surgery in CCTGA with the right ventricle as systemic ventricle has a suboptimal survival and limited freedom of reoperation. Death occurred mostly as a result of cardiac failure.

## Background

Congenitally corrected transposition of the great arteries (CCTGA) is a rare cardiac anomaly with an incidence of less than 1% of patients with congenital heart disease [1]. Characteristically the right atrium is connected to the morphologically left ventricle, which connects to the pulmonary artery and the left atrium is connected to the morphologically right ventricle, which connects to the aorta, resulting in atrio-ventricular discordance and ventriculo-arterial discordance, or double discordance [2]. In 90% of these patients associated anomalies are present as well, with ventricular septal defect as the most common, followed by pulmonary stenosis and atrial septal defect [1,3-5].

The prognosis of patients with CCTGA is variable with some patients showing satisfactory long-term survival [1,5-7]. However, both deteriorating right ventricular function on the long-term, as well as associated anomalies have an adverse effect on outcome. For instance a ventricular septal defect, pulmonary stenosis or arrhythmia have been found to limit the prognosis [6,8].

Repair with the right ventricle staying the systemic ventricle, including the Rastelli approach in case of subpulmonary obstruction [9], has for many years been conducted to correct CCTGA [2,5,10]. Ultimately, this often results in tricuspid valve regurgitation, dysfunction of the right ventricle and eventually heart failure [1,11,12]. In trying to improve this suboptimal outcome in CCTGA, the double-switch operation was introduced [6,8,13-15]. In this procedure, the left ventricle is incorporated as systemic ventricle and the right ventricle and tricuspid valve are no longer part of the systemic circulation. This approach is often referred to as being an anatomic repair [16-21]. Indeed, satisfying early and intermediate results were confirmed [22].

In a recent publication, however, no differences in the long-term survival rates between patients undergoing either repair could be found [17]. Furthermore, the superiority of the double-switch operation compared to the conventional repair could not be demonstrated in patients who had no tricuspid regurgitation before operation [17,23]. The finding of left ventricular dysfunction and issues related to new or residual valvular (aortic valve and tricuspid valve) incompetence and arrhythmias prevented the double switch operation (either with arterial switch or with conduit connection from right ventricle to pulmonary artery) to be labelled as the management of choice in CCTGA [24]. However, for patients who suffer from significant tricuspid valve regurgitation, the double switch repair is suggested to be an adequate treatment [17].

The purpose of this study was to present further results with regard to long term survival of repair in CCTGA with the right ventricle in the systemic circulation, in order to further contribute data in this challenging congenital anomaly.

## Materials and methods

### Patients

All 32 patients with CCTGA and two adequate ventricles who were surgically treated in the Erasmus MC between January 1972 and October 2008 were included in this series.

Detailed data of each patient were obtained from hospital records. All but four of the patients were under follow up in our centre. These four patients moved abroad and their data were censored at their last visit. The three patients with a cardiac transplantation were not included in the Kaplan-Meier analyses of survival and freedom from reoperation. The transplant procedures were done at ages 33, 34 and 47 years respectively for end-stage right ventricular failure in CCTGA.

The records were also analyzed for information on anatomy of the proximal coronary arteries. At the end of follow-up, dysfunction of the right ventricle and regurgitation of the tricuspid valve were graded subjectively as normal or mildly, moderately or severely reduced. Other variables included were New York Heart Association (NYHA) class (I, II, III or IV) and the need for a pacemaker.

### Statistical analysis

Data were analyzed using SPSS 15.0 for Windows (SPSS, Chicago, Il, USA). Patient survival rate and freedom from reoperation were analyzed using Kaplan-Meier curves. Cox regression analyses were used for analysing risk factors for mortality.

### Non-Rastelli group

The non-Rastelli group comprised 18 patients who were operated with preservation of the left ventricular outflow tract. In this group in the early part of the study 3 patients were treated with a pulmonary banding. The indication for surgery in this group was atrial septal defect in one patient, severe tricuspid valve regurgitation in four patients and ventricular septal defect (combined with atrial septal defect and pulmonary stenosis in three patients, with atrial septal defect in two patients, with atrial septal defect and pulmonary stenosis in one patient, with atrial septal defect, severe tricuspid valve regurgitation and pulmonary stenosis in one patient and with severe tricuspid valve regurgitation in one patient) in 13 patients. All ventricular septal defects were closed with a prosthetic patch. In 11 of the patients the tricuspid valve showed no regurgitation and in one a moderate regurgitation was left untouched. In the patients with pulmonary stenosis, this concerned valvular pulmonary stenosis and was treated with pulmonary valvotomy. In all six patients with severe tricuspid valve regurgitation the tricuspid valve was replaced with a prosthetic valve. Three patients had an Ebstein anomaly of the tricuspid valve. Two of them had severe regurgitation.

### Rastelli group

In 11 patients a Rastelli procedure was carried out. In six of these 11 patients a pulmonary arterial banding was done as a previous palliative procedure. In all of these patients a VSD was present. In 9 patients there was pulmonary stenosis and in 2 a pulmonary atresia. In 5 of them an ASD was present.

In all patients the ventricular septal defect was closed with a prosthetic patch and the pulmonary stenosis or atresia was treated with a conduit from the left ventricle to the pulmonary artery. Tricuspid valve regurgitation was diagnosed as moderate in one patient. No further anomalies were present in this group of patients.

### Cardiac transplantation

In three patients end-stage systemic ventricular dysfunction was the reason for cardiac transplantation at ages 33, 34 and 47 respectively. These three patients had an intact atrial and ventricular septum and an adequate subpulmonary outflow. One patient had a dextrocardia and a long history of cardiac failure before transplantation. The second patient also had a dextrocardia, with additionally mitral and aorta regurgitation, resulting in cardiac failure, finally leading to cardiac transplantation. The third patient had a pacemaker implantation for complete atrioventricular block, 11 years earlier, and suffered from end-stage right and left heart failure before undergoing cardiac transplantation.

## Results

The mean age at surgery for the 29 non-transplant patients was 13.8 years (sd 13.5 years, range 1 week - 48.7 years). This was 17 years (sd 16 years, range 1 week - 49 years) in the Non-Rastelli group, and 8 years (sd 5 years, range 2 - 17 years) in the Rastelli group. The mean follow-up period was 11.5 years (sd 9.8 years, range 7 days - 32.0 years).

### Coronary anatomy

In 13 out of the 32 patients information on coronary anatomy was explicitly available. In one patient a circumflex coronary artery arose from the right coronary artery. In two patients a single coronary orifice was described. In two patients a coronary branch crossed the subpulmonary outflow tract. In eight patients the coronary arteries were described as fitting with CCTGA. This means that, connected to the right posterior aortic sinus, the right-sided left coronary artery with its left anterior descending and circumflex branches supplies the right-sided left ventricle and, connected to the left posterior aortic sinus, the right coronary artery with its posterior descending branch provides the left-sided right ventricle.

In 19 patients, information on coronary arterial anatomy was not described, and original catheterization films were no longer available.

### Pacemaker

Total atrioventricular block, with pacemaker insertion, occurred in two patients prior to cardiac surgery (one in the later non-Rastelli group, one in the later Rastelli group).

In the early part of the series, surgery related atrioventricular block, necessitating implantation of a permanent pacemaker occurred in seven patients (six in the non-Rastelli group, one in the Rastelli group).

Postoperative atrioventricular block necessitating a permanent pacemaker occurred in an additional three patients (two in the non-Rastelli group, two and 16 years after surgery and one in the Rastelli group, three years after surgery).

At a medium of 12 years (range 7 days - 32 years) of follow up a total of 12 out of 29 patients (41%) had a permanent pacemaker.

No significant difference between the groups was found with regard to pacemaker implantation.

### Tricuspid valve regurgitation

In six patients the tricuspid valve was replaced at the primary procedure (all in the non-Rastelli group). In two patients the moderate tricuspid valve regurgitation was left untouched in the non-Rastelli group. In an additional nine of the remaining 20 patients, moderate to severe tricuspid valve regurgitation developed during a mean follow up of 10 years (sd 9 years, range 1 month - 24 years). In four patients (all in the non-Rastelli group) the tricuspid valve was replaced at a median of 15 years (sd 10 years, range 2 - 24 years) after primary surgery.

Tricuspid valve regurgitation was more often seen in the non-Rastelli group. We found no correlation with right ventricular failure.

### Right ventricular failure

At the end of follow-up 14 (seven in the non-Rastelli group, seven in the Rastelli group) of the 20 patients were suffering from right-ventricular dysfunction, in 12 of them (six in the non-Rastelli group, six in the Rastelli group) progressively, resulting in 10 patients (six in the non-Rastelli group, four in the Rastelli group) with moderate to severe failure. In 4 of these 14 patients (two in the non-Rastelli group and two in the Rastelli group), there was mild right ventricular dysfunction. In 6 patients there was normal right ventricular function. In three of these 6 patients (two in the non-Rastelli group, one in the Rastelli group) failure was diagnosed at presentation, but after surgery the function of the right ventricle improved to normal.

Surprisingly, the NYHA class at the end of follow-up of these 20 patients was found to be NYHA I in 11 patients. Five patients were in NYHA class II, three of these patients suffered moderate ventricular failure and in the other two patients no right ventricular failure was found at rest. One patient was in NYHA class III. Unfortunately, in three patients no information with regard to their NYHA class was available.

The three patients who were treated with cardiac transplantation all had a severely failing right ventricle resulting in severe shortness of breath, classified as NYHA class III before transplantation.

### Mortality

Nine patients in our series died, two early and seven during follow up.

Early mortality was due to sepsis after a non-Rastelli procedure in one patient and, in the early part of the series, to cardiac failure associated with atrioventricular block in another non-Rastelli patient.

Seven patients died during follow up (four in the non-Rastelli group and three in the Rastelli group). In the non-Rastelli group a patient of seven years old died 10 months after surgery following a pacemaker implantation in relation to anoxia with resulting neurological damage. Another patient of seven years old died three years after surgery due to congestive heart failure with a failing right ventricle. A 19 years old patient died 5.5 years after surgery from end-stage cardiac failure. A 35 years old patient died 23.5 years after surgery due to progressive cardiac failure and pneumonia.

In the Rastelli group one patient of 3 years old died of pneumosepsis, confirmed at autopsy, 5 months after the procedure. Two patients died 11 years and 14 years after surgery, unfortunately no details on mode of death are available.

**Figure 1:**
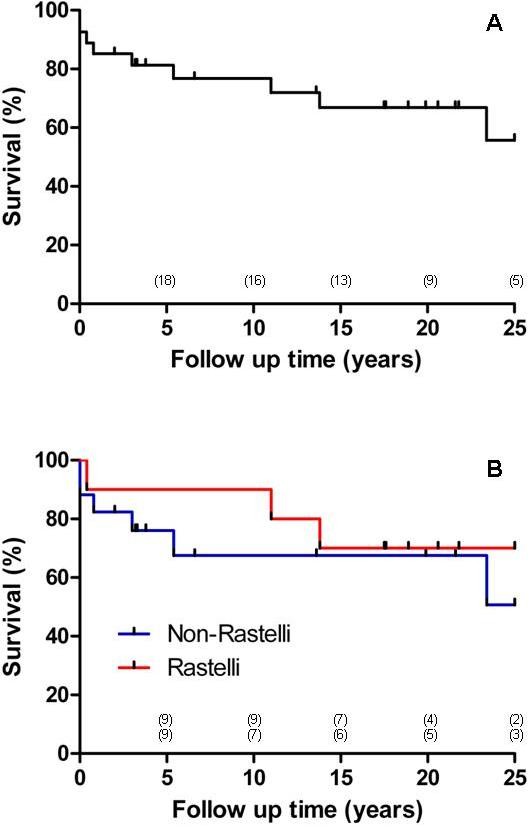
**Kaplan-Meier survival after surgery**. A) Overall survival. B) Survival split by non-Rastelli and Rastelli surgery. Between brackets the number of patients at risk.

Survival rates of the patients in our series are represented in Figure 1. The survival at 10, 20 and 30 years after surgery was 74% (CI 65-83%), 63% (CI 53-73%) and 52% (CI 38-66%) respectively. For the non-Rastelli group these data at 10, 20 and 25 years were 62% (CI 48-76%), 62% (CI 48-76%) and 42% (CI 23-61%) respectively. For the Rastelli group the data at 10, 20 and 25 years were 89% (CI 78-100%), 67% (CI 51-83%), and 67% (CI 51-83%) respectively.

### Reoperation

In 12 patients reoperations were done. These were done a mean of 10 years (sd 8 years, range 0.7 - 24 years) after primary surgery.

In the non-Rastelli group six patients were reoperated after a mean of 11 years (sd 11, range 0.7 - 26 years). In five of them newly developed tricuspid valve regurgitation was treated with replacement of the tricuspid valve with a prosthetic valve. In one of these patients a central atrial septal defect was closed, that had been left open at primary surgery. In another one of these patients a residual ventricular septal defect was closed as well.

In the Rastelli group six patients were reoperated after a mean of 9 years (sd 7, range 0.7 - 19 years). In all six of them a conduit replacement was carried out, combined with tricuspid valve replacement for newly developed tricuspid valve regurgitation in two patients and combined with closure of a residual ventricular septal defect in three patients. In four patients a second conduit replacement was done.

**Figure 2:**
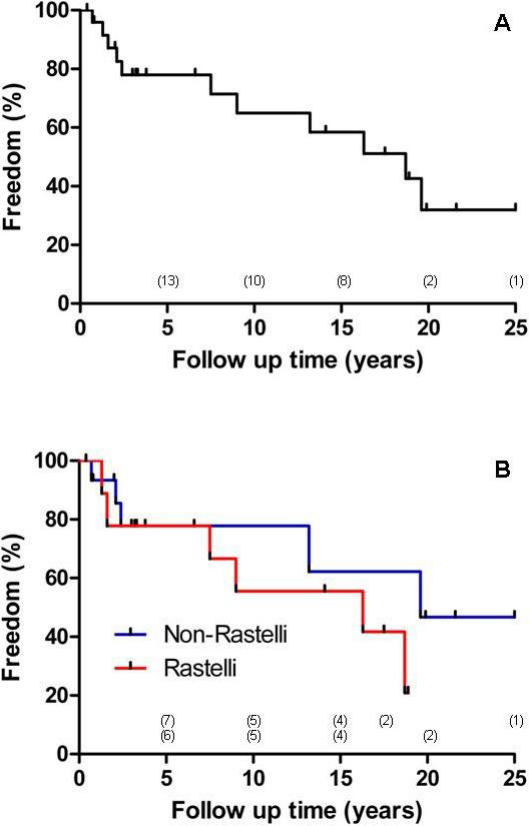
**Freedom of reoperation after primary surgery**. A). Overall freedom of reoperation. B) Freedom of reoperation split by non-Rastelli and Rastelli surgery. Between brackets the number of patients at risk.

Freedom of reoperation was found to be 65% (CI 54-76%), 32% (CI 19-45%) and 32% (CI 19-45%) after 10, 20 and 25 years respectively in the overall group. In the non-Rastelli group the freedom of reoperation at 10, 20 and 25 years was 78% (CI 56-100%), 47% (CI 11-83%) and 47% (CI 11-83%) respectively. In the Rastelli group the freedom of reoperation was 56% (CI 23-88%) and 21% (CI 0-54%) after 10 and almost 19 years respectively. The freedom of reoperation is depicted in Figure 2.

## Discussion

Survival after surgical repair in CCTGA with a right ventricle as systemic ventricle has been evaluated in different studies [1,5,7,11,25]. The mid-term results are often reported as satisfactory. Some studies describe no significant changes in right ventricular ejection fraction or limitation of exercise intolerance over an observation period of 10 years [25]. However, others state that the prognosis in CCTGA mainly depends on the presence of associated anomalies, on significant tricuspid valve regurgitation or on right ventricular dysfunction [5,7,11]. The quality of life in this regard may be limited due to diminished exercise performance and deterioration of NYHA class and may lead to a 50% mortality due to right ventricular failure at a mean age of 38.5 years (sd 12.5) [1].

The overall survival in our study does not differ significantly from other studies. A 20-year survival of 48 to 75% has been reported [5,26,27]. In our series the 20-year overall survival was 63%. Shin'oka et al. [17] had a 32-year survival of 62.4% in their non-Rastelli group and 78.5% after 27 years in their Rastelli group. Our results show a survival at 25 years after surgery of 42% in the non-Rastelli group and of 67% in the Rastelli group (Figure 1).

Significant risk factors for right ventricular dysfunction over time were found to be tricuspid valve regurgitation, complete atrioventricular block, the need for pacemaker therapy and arrhythmias [5,8,11,12]. Deteriorated right ventricular function was diagnosed in 56% of 45-year old patients with CCTGA and with associated anomalies [8]. In surgical repair in CCTGA with the right ventricle as systemic ventricle, an association can be recognized between right ventricular dysfunction and suboptimal results.

The expected better systemic ventricular function was the reason for the pursuit of anatomic repair [13]. In the short-and midterm follow-up of the double switch procedure for CCTGA a reduction of complications was shown [13,20,22,28]. However, different studies in patients undergoing anatomic repair procedures could not show a reduction of systemic ventricular dysfunction [14,16,17,24]. Understandably, an uncertainty on the value of anatomic repair in CCTGA emerged. Due to left ventricular dysfunction, to new or residual valvular dysfunction of the aortic and tricuspid valve and to arrhythmias, anatomic repair could not be labelled as the ideal management option in CCTGA [24]. In addition, the incidence of heart block was reported to be higher in the anatomic repair group resulting in more pacemaker implants [17,22,24].

In case of severe tricuspid valve regurgitation, the anatomic repair is more likely to increase the survival rate than conventional repair [12,17]. However, when no tricuspid valve regurgitation is present preoperatively, a survival rate of even 72% at 30 years can be reached with conventional repair [17].

In this regard, tricuspid valve regurgitation occurs frequently in patients with CCTGA at long-term follow-up after conventional repair [3,7,11]. In patients with normal tricuspid valve function in CCTGA, undergoing conventional biventricular repair without any intervention on the tricuspid valve, 52 to 67% developed moderate or severe regurgitation after three to 10 years of follow-up [5,12,28]. However, others found that only a morphologically abnormal tricuspid valve was significantly associated with occurrence of tricuspid valve regurgitation and that only 26% of the patients had increasing tricuspid valve regurgitation, following often early after open-heart surgery within a follow up of 12 years [11].

In some series tricuspid valve regurgitation at follow-up was a predictor for reoperation [17], but this could not be confirmed in the present series or by others [18]. Overall freedom from reoperation was reported to be 80% at five years and 64% after 32 years in conventional groups and 97 at five years and 77% after 27 years in a Rastelli group [17]. After double switch operation 86% freedom of reintervention at five years is reported [18]. Our results fit well with these data, the early results being more promising then the long-term outcome. Especially when tricuspid valve regurgitation or abnormalities are diagnosed, anatomic repair can be considered above conventional procedures [5,17].

To accomplish a retraining of the involved left ventricle in preparation for a double switch procedure, a preparatory banding of the pulmonary artery has been applied. In the double switch procedure, this is considered to increase the risk of deterioration of the function of the morphologically left ventricle over time compared to patients whose ventricle does not require training [29]. Whether or not this is related to limited capacity for remodelling of the myocardium, to abnormal coronary arterial anatomy, to limited coronary arterial adaptation capacity or to other factors is yet unknown. However, an important finding is the abnormal pattern of coronary arterial anatomy in CCTGA [30]. Although the information in our study was incomplete, a word of caution may be relevant with regard to an increased incidence of abnormal anatomy of the proximal coronary arteries.

### Limitations of this study

In general, CCTGA has a low incidence and a variable presentation, which complicates grouping of data. In addition, surgical procedures and standards have changed over time and have different periods of follow-up observations. Therefore, abstractions on patient outcome and results should be interpreted with caution. Our study is retrospective in nature and unfortunately no quantitative data were available on systemic ventricular function.

## Conclusions

Our series confirms that in long term follow up, surgery in CCTGA with the right ventricle as systemic ventricle has a suboptimal survival and limited freedom of reoperation. There is an increased incidence of abnormal anatomy of the proximal coronary arteries. An important number of patients will need tricuspid valve replacement at either primary or later surgery. An important number of patients will need a pacemaker at any stage of observation. Death occurred mostly as a result of cardiac failure.

## Competing interests

The authors declare that they have no competing interests.

## Authors' contributions

AJJCB - Study supervision, data interpretation, drafting manuscript

SJH - Data collection, statistical analysis, drafting manuscript

PLJ - Conception and design, drafting manuscript

MW - Conception and design, data interpretation

APK - Study supervision, statistical analysis

All authors have read and approved the final manuscript.
